# Evolution of Canada’s Boreal Forest Spatial Patterns as Seen from Space

**DOI:** 10.1371/journal.pone.0157736

**Published:** 2016-07-06

**Authors:** Paul D. Pickell, Nicholas C. Coops, Sarah E. Gergel, David W. Andison, Peter L. Marshall

**Affiliations:** 1 Faculty of Forestry, University of British Columbia, Vancouver, British Columbia, Canada; 2 Bandaloop Landscape-Ecosystem Services, North Vancouver, British Columbia, Canada; University of Guelph, CANADA

## Abstract

Understanding the development of landscape patterns over broad spatial and temporal scales is a major contribution to ecological sciences and is a critical area of research for forested land management. Boreal forests represent an excellent case study for such research because these forests have undergone significant changes over recent decades. We analyzed the temporal trends of four widely-used landscape pattern indices for boreal forests of Canada: forest cover, largest forest patch index, forest edge density, and core (interior) forest cover. The indices were computed over landscape extents ranging from 5,000 ha (n = 18,185) to 50,000 ha (n = 1,662) and across nine major ecozones of Canada. We used 26 years of Landsat satellite imagery to derive annualized trends of the landscape pattern indices. The largest declines in forest cover, largest forest patch index, and core forest cover were observed in the Boreal Shield, Boreal Plain, and Boreal Cordillera ecozones. Forest edge density increased at all landscape extents for all ecozones. Rapidly changing landscapes, defined as the 90^th^ percentile of forest cover change, were among the most forested initially and were characterized by four times greater decrease in largest forest patch index, three times greater increase in forest edge density, and four times greater decrease in core forest cover compared with all 50,000 ha landscapes. Moreover, approximately 18% of all 50,000 ha landscapes did not change due to a lack of disturbance. The pattern database results provide important context for forest management agencies committed to implementing ecosystem-based management strategies.

## Introduction

The relationship between ecological processes and spatiotemporal heterogeneity has long been a critical topic of ecological research [1,2]. Landscape pattern indices have been developed to characterize valuable ecological patterns [3]. For example, fragmentation of forest cover captures habitat loss, habitat perforation, and connectivity [4]. As a consequence, the spatial and temporal context of landscape patterns has emerged as a research priority in conservation science [5].

Characterizing patterns associated with landscapes present challenges for applying rigorous statistical analyses: spatial data of sufficient quality is not always available or consistent across jurisdictions; samples tend to be autocorrelated (*i*.*e*., not independent); and landscapes are unique natural experiments that are challenging to replicate. Several solutions have been proposed to overcome these limitations including the development of comprehensive databases of landscape patterns from satellite imagery [5]. Large area land cover maps have already been utilized for fragmentation analyses in Canada [6] and the United States [5]; however, these analyses have been limited to a single time period.

Advancements in automated remote sensing techniques have provided the means to map large areas from regional to global scales [7,8]. Large area mapping of forest cover changes has resulted in new opportunities to investigate the relationship between disturbance processes and spatiotemporal heterogeneity [7,9,10,11,12,13]. Forest cover maps provide a unique opportunity to understand how patterns of landscape structure vary spatially and the potential consequences of changing landscape structure for habitat availability and loss. For example, the METALAND project represents the largest database of landscape pattern indices derived from the *circa* 1992 National Land Cover Database for the conterminous United States [5]. Similarly, the work by Wulder *et al*. [6,14] utilized the Earth Observation for Sustainable Development of Forests land cover map of Canada to summarize fragmentation of boreal forests *circa* 2000. Such research enhances the ability to identify representative landscapes across broad scales and to characterize the spatial and statistical context of landscape pattern indices [5,15]. The behavior of landscape patterns remains to be characterized for large areas through time and at multiple scales.

Some of the questions initially put forward by Cardille *et al*. [5] about the spatial, temporal, and statistical context of landscape pattern indices stemmed from limited access to comprehensive historical data at the time. Since 2008, the entire Landsat archive has been opened with free access [16], which has significantly advanced progress in time series analysis over the current decade. Access to new data and technology allows us to answer a very basic, but pressing question in spatial ecology: How have spatial patterns changed over time?

Boreal forests represent good opportunities for testing hypotheses about temporal changes in landscape patterns because these forests are easily distinguished from other non-forest land cover classes. More research is needed in the area of spatial landscape pattern change in order to better understand the impacts on the flow of a wide range of ecosystem goods and services such as habitat, carbon sequestration, and wood fibre [17]. Moreover, boreal forests are particularly sensitive to climate change and transitions to alternative assemblages because most boreal tree genera are temperature-limited [18,19]. Large even-aged patches of forest are emergent keystone landscape structures in boreal forests as a consequence of the inverse relationship between fire size and frequency [20,21]. Yet there is also significant structural and compositional diversity at finer scales that is readily observed by remotely sensed imagery [22,23,24]. Lastly, intensive resource extraction has created, in some areas, a novel overlay of structures in addition to various sources of intrinsic variation [22,25].

The degree to which boreal landscape patterns have changed in recent years is largely unknown and is of great interest to Canadian resource managers planning resource extraction to emulate historical landscape conditions [26,27]. This approach to resource development is the cornerstone of ecosystem-based management (EBM) and has been implemented in several areas in the boreal forest across Canada (*e*.*g*., [22]). The rationale is that historical landscape conditions maintained the recent biodiversity and habitat for a wide range of species [27,28]. Delimiting the baseline condition, known as the historical range-of-variability, is a requisite for EBM implementation [26,29]. The historical range-of-variability can be used as a benchmark to assess current landscape condition by providing a population of landscapes that is suitable for robust statistical analysis [5]. Such a benchmark is also necessary as the baseline for creating models for detecting deviations from the historical range-of-variability due to climate change, as well as predicting future landscape conditions under different climate scenarios [30].

In this paper, a methodology is introduced for the enumeration of a landscape pattern database for the boreal forest of Canada derived from time series Landsat satellite imagery. The primary objective was to quantify the recent high-magnitude changes to the forest landscape patterns and populate a database of landscape pattern indices that could be used to assess the historical trends of boreal forest landscape patterns in Canada. Finally, we demonstrate how the database and indicators can be used together to identify regions of the boreal forest undergoing significant changes to forest cover abundance and configuration.

## Materials and Methods

### Sampling design and data acquisition

**Fig 1:**
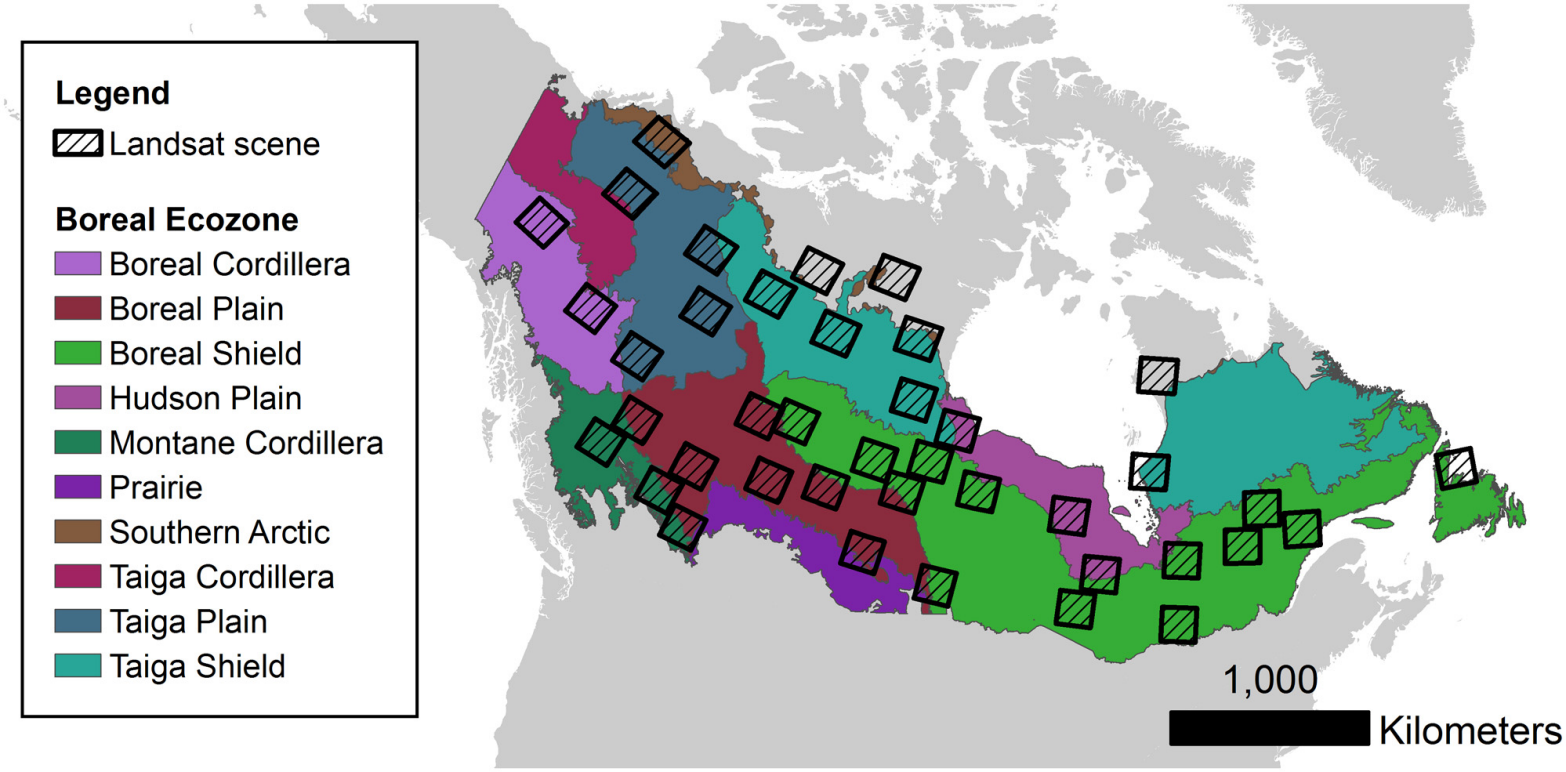
Sample of Landsat tiles distributed across major ecozones of Canada [57].

A sampling frame was defined for the Canadian boreal zone [31] based on intersecting WRS-2 Landsat path-rows (hereafter referred to as tiles) and forest cover derived from Sexton et al. [32]. Tiles were ranked based on the global continuous tree cover fields developed by Sexton and colleagues [32] and tiles ranked above the 5^th^ percentile of forest cover were included in the population sampling frame. A final random sample of *n* = 40 tiles were stratified by major bio-climatic zones [33] within the Canadian boreal zone [31] (Fig 1). All Landsat images were acquired during the growing season and neighbor overlapping scenes with less than 70% cloud cover were downloaded from the archive for each targeted tile. The growing season was defined uniquely for each Landsat tile using two attributes: (1) length-of-season was derived from the scene center latitude of each Landsat tile regressed on empirically-derived length-of-season for northern latitudes based on [34]; and (2) the start- and end-of-season days defined equally distant from July 28, which was considered the peak normalized differenced vegetation index (NDVI) day of year [35]. Defined in this manner, the start-of-season varied from May 7 to June 15 and the end-of-season ranged from September 9 to October 18 depending on the latitude of the Landsat tile.

Each pixel from all images in a given year was scored based on best available pixel (BAP) criteria: imaging sensor (Thematic Mapper or Enhanced Thematic Mapper Plus); cloud shadow and proximity to cloud and cloud shadow as a Gaussian function of a 50 m buffer; day of year as a Gaussian function of the growing season; and atmospheric opacity [36]. Pixels with the highest scores were then composited into annual BAP images for the change detection procedure described in the next section. For some years and locations, there was no best available pixel due to data gaps on all of the available images. In these cases, the spectral information was interpolated between the most two recent years with data. Pixel time series with three consecutive years of no data or with more than six total years of no data were excluded from further analysis.

### Mapping forest cover changes

The process for mapping forest cover changes involved training an annual forest mask and then applying spectral trend logic to the trajectory of each pixel to derive the pixel state. There were three possible states for each pixel: persisting forest, persisting non-forest, and forest cover change. Persisting forest and persisting non-forest indicated that the pixel was forested or non-forested for all annual images. Forest cover changes were characterized by the change of a pixel from a forested state to a non-forested state and vice-versa. The forest cover changes were mapped during the study period (1985 to 2010) using a modified version of the Vegetation Change Tracker (VCT, [37]) for each target Landsat tile. Previous studies have evaluated the accuracy of the VCT across a wide range of forest types including boreal forests and concluded that the VCT generally underestimates disturbance (*i*.*e*., higher omission error rate) with the majority of disturbance events correctly identified to within ±1 year [38,39]. Overall accuracies for the VCT using change and no change categories have been reported to exceed 90% [38,39].

Forest cover masks were trained using the Moderate-resolution Imaging Spectroradiometer (MODIS) Vegetation Continuous Fields (MOD44B) product from the year 2000 [32], annual NDVI values, and visual interpretation of annual true-color images. The disturbance index (DI) [40] was used for mapping forest cover changes with thresholds that were developed specifically for boreal forests [39]. The DI is a linear combination of the brightness, wetness, and greenness components of the Tasselled-Cap Transformation [40,41]. Pragmatically, DI values that deviate significantly from zero (*i*.*e*., positively or negatively) are more likely to be non-forested [40]. Pixels that exceeded the DI threshold for at least three consecutive years with a DI change magnitude greater than five were labeled as *changed*. This procedure ensured that only the highest magnitude disturbed pixels were considered and reduced the occurrence of stress or changes in vegetation condition being mapped as forest disturbance [42]. This magnitude threshold attenuated the extent of many low and mixed-severity fires. Pixels that consistently exceeded the DI thresholds for the length of the time series were labeled as *persisting non-forest* and pixels that were within the DI thresholds were labeled as *persisting forest*. The final forest cover maps were filtered to a minimum mapping unit (MMU) of one ha using the eight-neighbor pixel rule to ensure that the effect of map misclassification errors on landscape pattern indices was minimized [43]. Non-forest patches smaller than the MMU were reclassified as forest and forest patches smaller than the MMU were reclassified as non-forest. Pixels disturbed in multiple years were relatively infrequent during the 26-year time series and generally below the target MMU of one ha, so for simplicity only the most recent disturbances were considered in the analysis.

In order to account for forest cover gains over time, forest recovery was defined spectrally to be consistent with the forest disturbances. Using the disturbance maps produced by the VCT, forest recovery was defined as 80% of the mean Normalized Burn Ratio value from the two years before the disturbance. The Normalized Burn Ratio was selected because it is a robust recovery indicator compared to other spectral indices and likely represents the structural components of vegetation [44,45]. The recovery trends were computed using the annual BAP composite images containing the original spectral information. The dynamic recovery model ensured that landscape pattern indices would not be biased in later years due to cumulative forest cover losses (*e*.*g*., over-estimating landscape fragmentation).

### Quantifying landscape patterns

FRAGSTATS (v4.2.598) was used to compute four different landscape pattern indices (Table 1) for every annual forest cover map in the sample [46]. The landscape pattern indices were only computed for forest patches. The indices were selected because they are commonly computed, provide generic, useful patterns, and are comparable across scales [2,47]. The ability of indicators to measure across scales is particularly important because the scales at which significant patterns emerge are not always obvious [48], and thus are largely identified through exploratory analyses. For the boreal forest, a range of scales that are potentially useful for reporting national and continental fragmentation indices remain to be tested [6]. For this study, the pattern indices defined in Table 1 were calculated at four scales by varying landscape extent using a grid of landscapes with the native grain size of the Landsat imagery (0.09 ha). The scales were of approximate map extents: 50,000 ha; 25,000 ha; 10,000 ha; and 5,000 ha. To ensure that trends were only assessed for the boreal zone and that data gaps (*i*.*e*., background) did not adversely affect the pattern indices, only landscapes with 25% or less background and 10% or more of the boreal zone were included in the final tabulations. Landscapes with significant agricultural influence (*i*.*e*., > 1%) per the Canadian Agricultural Inventory database [49] were excluded from the analysis. Core forest indices were calculated based on a 120 m depth from the edge of forest patches representing four Landsat pixels, which was consistent with known edge effects in boreal forests [50].

**Table 1:** Landscape pattern indices that were calculated on an annual basis of forest cover.

Index	Formulation (units)	Interpretation	Relevance in boreal forests	References
Forest cover	Total forested area divided by total landscape area multiplied by 100 (%).	A measure of the relative coverage of forest with units that are standardized to landscape extent. Higher values indicate more forest cover relative to non-forest cover classes.	Relates to forest dominance.	[51,52]
Largest forest patch index	Area of the largest patch in the landscape divided by total landscape area multiplied by 100 (%).	A measure of the relative contribution of the largest forest patch to the landscape with units that are standardized to landscape extent. Higher values indicate lower fragmentation.	Relates to forest intactness and forest connectivity.	[46]
Forest edge density	Total perimeter of forest patches divided by total landscape area (m per ha).	A measure of all forest edges in a landscape with units that are standardized to landscape extent. Higher forest edge density indicates higher fragmentation.	Relates to abundance of edge habitat, predator-prey dynamics, and other ecological responses at edges.	[53]
Core forest cover	Total core area divided by total landscape area multiplied by 100 (%).	A measure of the relative coverage of core forested area with units that are standardized to landscape extent. Higher values indicate lower fragmentation.	Relates to edge impacts on forests and the relative abundance of core forest habitat.	[46]

Autocorrelation was assessed both spatially and temporally for all four pattern indices to provide explicit measures of spatial and temporal distances at which two landscapes could be considered independent samples for statistical analyses [54]. Latitude and longitude coordinates were calculated for the centroid of every landscape at all extents. Spatial autocorrelation was measured at each extent between the centroid coordinates of the landscapes using Moran’s I index of autocorrelation [55]. Only lag distances with at least 1,000 paired landscapes were considered in the spatial autocorrelogram. The large-lag standard error multiplied by two was computed for each lag class to assess the significance of Moran’s I at α = 0.05 for each lag distance. Temporal autocorrelation was computed for the time series of every landscape at annual lag intervals to provide an estimate of the temporal autocorrelation present in the data [56]. Significance of temporal autocorrelation was assessed using boxplots with interquartile range outside two standard errors of the mean correlation value.

Temporal trends in the pattern indices were first tested for monotonicity (*i*.*e*., unidirectional positive or negative change trend) for each 50,000 ha landscape and for major ecozones [57] using non-parametric Mann-Kendall (M-K) tests with the Kendall R package [58]. The M-K test is robust and performs with higher power for non-normal data compared with standard parametric models [59]. Confidence bounds (95%) were calculated for the M-K test statistic (Tau) using a block bootstrap method with 500 repetitions in order to account for potential bias caused by autocorrelation. Theil-Sen lines were then fitted to the monotonic trends using the openair R package [60], which are robust with non-normal data and outliers compared with using standard linear regression models [61]. The slope and intercept of the Theil-Sen lines were calculated as the median slope of all pairwise point comparisons of the serial data. Median forest cover was computed annually for each ecozone and these serial data were tested for monotonic trends using the M-K test. All statistical tests were performed at the significance level α = 0.05.

### Distinguishing landscape change

We distinguished between three classes of landscape pattern change for the 50,000 ha landscapes based on relative forest cover change: (1) high magnitude change; (2) moderate magnitude change; and (3) no change. The significance of M-K tests performed on the slopes of forest cover over the study period for individual 50,000 ha landscapes was used as a benchmark to determine which landscapes were changing. Landscapes that did not have significant slopes for forest cover change over time were considered *no change*. Landscapes that had significant monotonic slopes were partitioned into *moderate magnitude change* if the change slope was below the 90^th^ percentile and *high magnitude change* if the change slope was above the 90^th^ percentile.

## Results

First, we present overall trends and emergent properties of the indices for all the extents and ecozones. We then present results of spatial and temporal autocorrelation. Finally, we present trends of boreal forest spatial patterns in landscapes undergoing forest cover changes and the trends of change variation among landscapes over time.

### Overview

Approximately 72 Mha of the Canadian boreal zone was analyzed. Permanent water accounted for more than 8.3 Mha of the sampled boreal zone. Within the sampled boreal zone, persisting forest and persisting non-forest (from 1985 to 2010) accounted for approximately 77% (49.2 Mha) and 18% (11.7 Mha) of the non-water area, respectively. Approximately 4.4% (2.8 Mha) of the non-water area or about 5.4% of the forested area was disturbed at some point during the study period of 1985 to 2010. In total, approximately 82% (52 Mha) of the non-water area was forested at some point during the time series.

**Fig 2:**
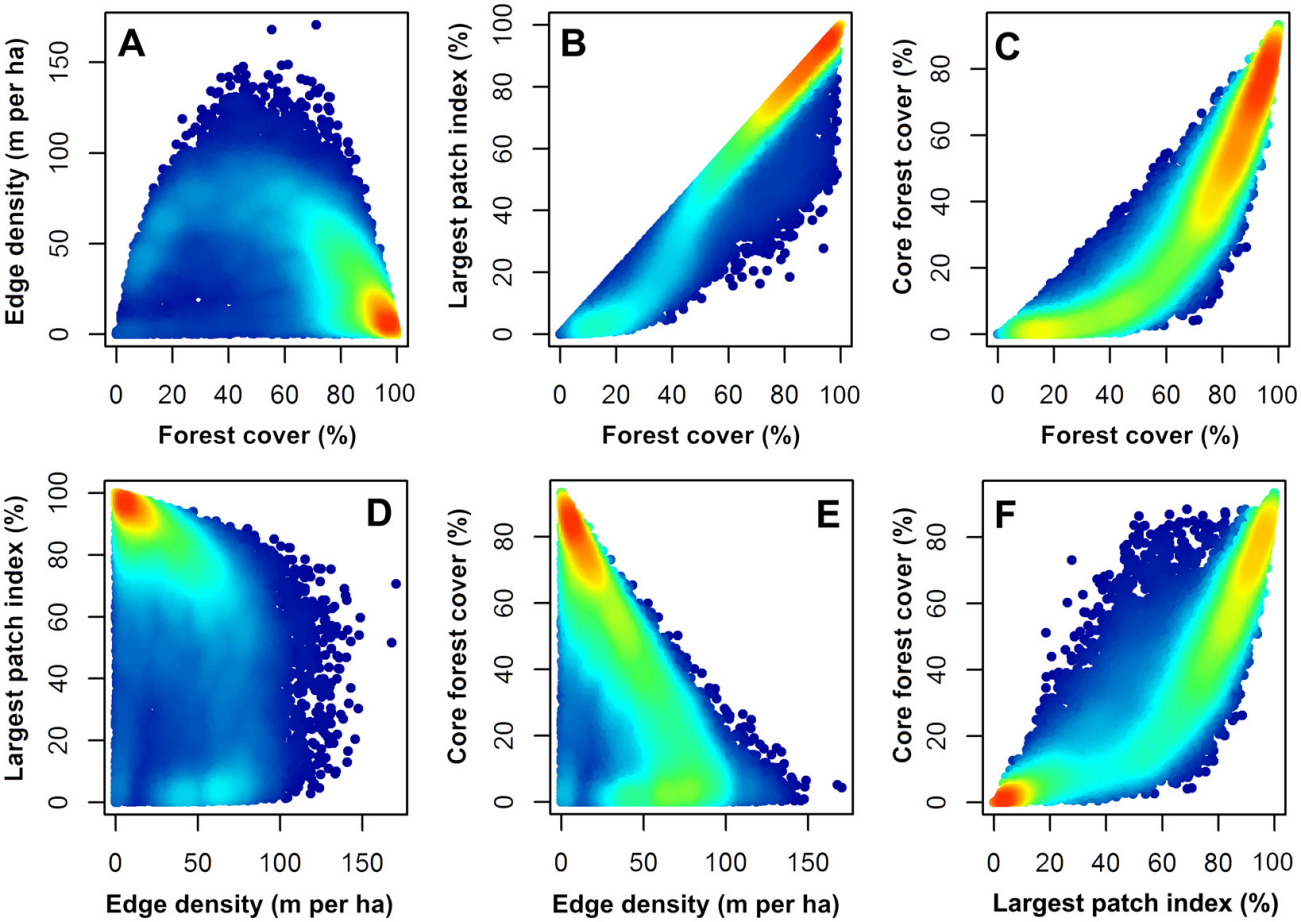
Heat-map scatterplots showing the relationships between the four landscape pattern indices in 2010 for the 5,000 ha landscapes (*n* = 18,185).

The total number of landscapes that met the selection criteria (*i*.*e*., ≤ 25% background data and ≥10% of the intersecting boreal zone) were distributed as follows across the extents of analysis: 18,185 landscapes at the 5,000 ha extent; 9,087 landscapes at the 10,000 ha extent; 3,451 landscapes at the 25,000 ha extent; and 1,662 landscapes at the 50,000 ha extent. Heat map scatterplots showing the relationships between the four landscape pattern indices are given in Fig 2. The scatterplots of the indices indicate mostly non-linear relationships, with some distinct aggregations in pattern and trajectories. For example, landscapes with high forest cover and largest patch index tend to have low edge density (Fig 2a and 2d). Other relationships among the indices indicated two basins of attraction with well-defined trajectories (Fig 2b, 2c, 2e and 2f). For example, landscapes tended to have either high or low core forest cover and this was primarily related to the overall proportion of forest cover (Fig 2c) and the largest patch index (Fig 2f). Both core forest cover and largest patch index were directly related to, and limited by, forest cover (Fig 2b and 2c) because these indices can never exceed the total forest cover. Variability of edge density increased as core forest cover declined (Fig 2e).

### Autocorrelation of landscape pattern indices

**Fig 3:**
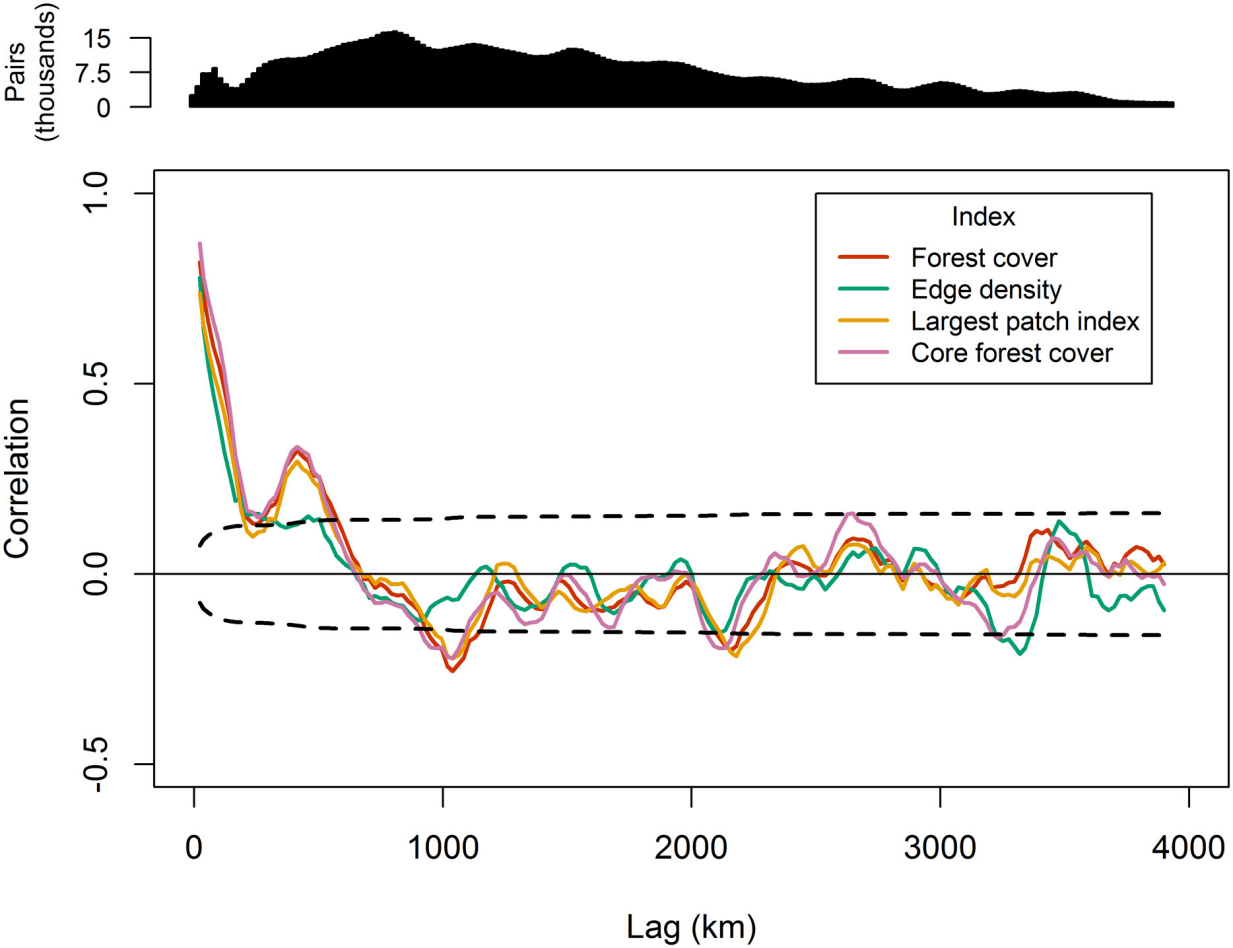
Spatial correlograms of the four landscape pattern indices observed in 2010 for the 50,000 ha landscapes. The dashed black lines indicate lag-large standard error bars for correlation (*i*.*e*., 95% confidence bounds). Only lag distances with at least 1,000 paired landscapes are shown.

**Fig 4:**
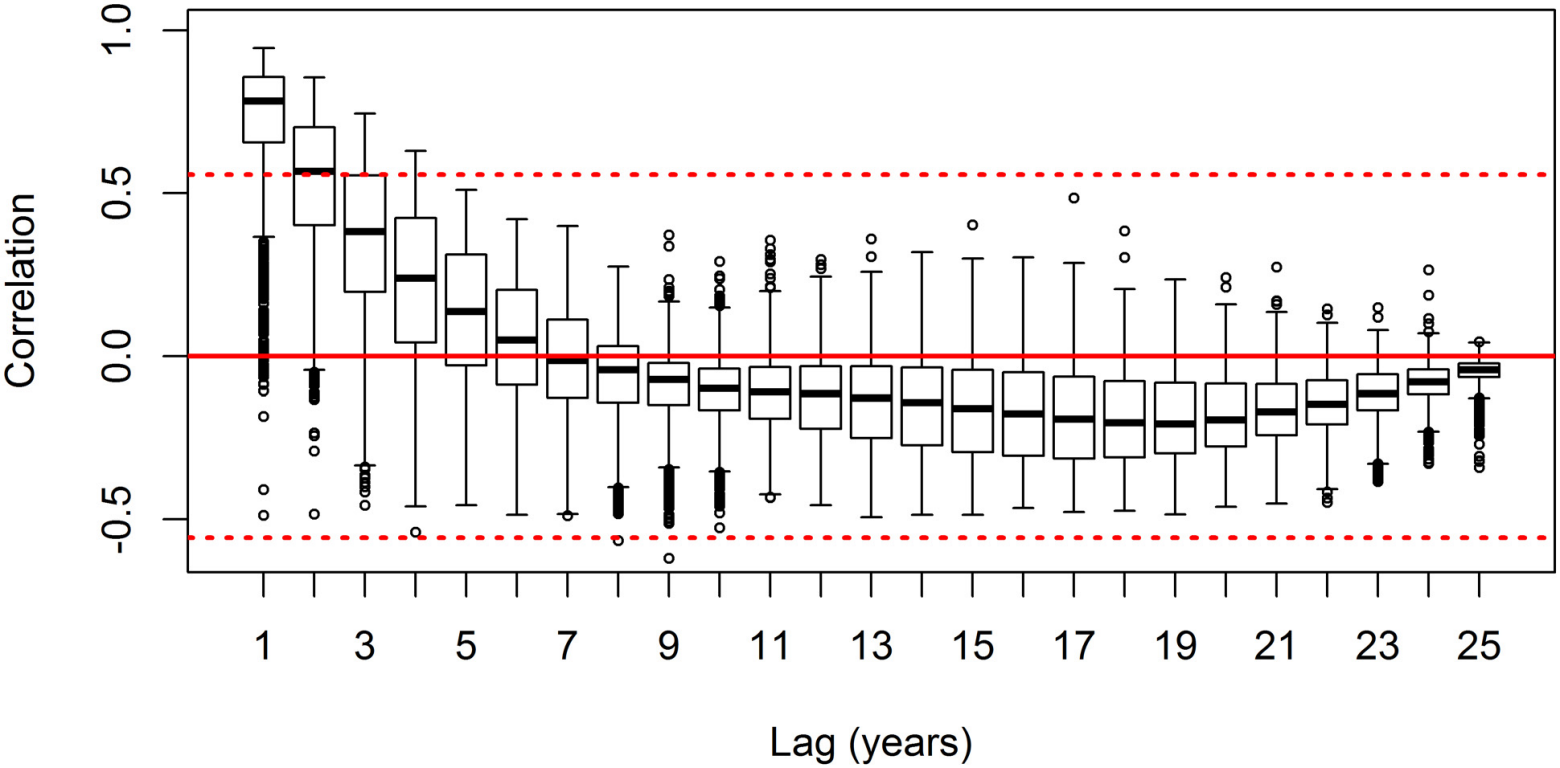
Temporal correlograms of forest cover observed for the 50,000 ha landscape extent. Each boxplot has *n* = 1,662 landscapes and represents the distribution of autocorrelation values at each lag. Solid red line is correlation = 0. Dashed red lines represent ±2 standard errors of the mean correlation value (*i*.*e*., 95% confidence bounds). Lags with interquartile range outside ±2 standard errors are considered significant for all 50,000 ha landscapes.

Spatial correlograms of landscape pattern indices for the 50,000 ha extent in 2010 are presented in Fig 3. High autocorrelation was observed up to approximately 190 km (Fig 3), which generally represented the greatest distance between landscapes within the same Landsat tile. A second peak in positive autocorrelation was observed for forest cover, largest patch index, and core forest cover at approximately 413 km, which was followed by a negative peak at approximately 1,039 km (Fig 3). A repeating cyclical pattern in autocorrelation was observed for lag distances greater than 682 km (*i*.*e*., the *x*-intercept) (Fig 3). Together, these observations indicated a non-monotonic trend in spatial autocorrelation and the emergence of hole-effect behavior in the spatial autocorrelation structure of the landscape pattern indices [62]. Forest cover was significantly correlated for up to two years as indicated by boxplots with interquartile range outside two standard errors of the mean correlation value (Fig 4). At temporal periods greater than two years, the autocorrelation was no greater than what would be expected by chance alone.

### Forest cover

**Fig 5:**
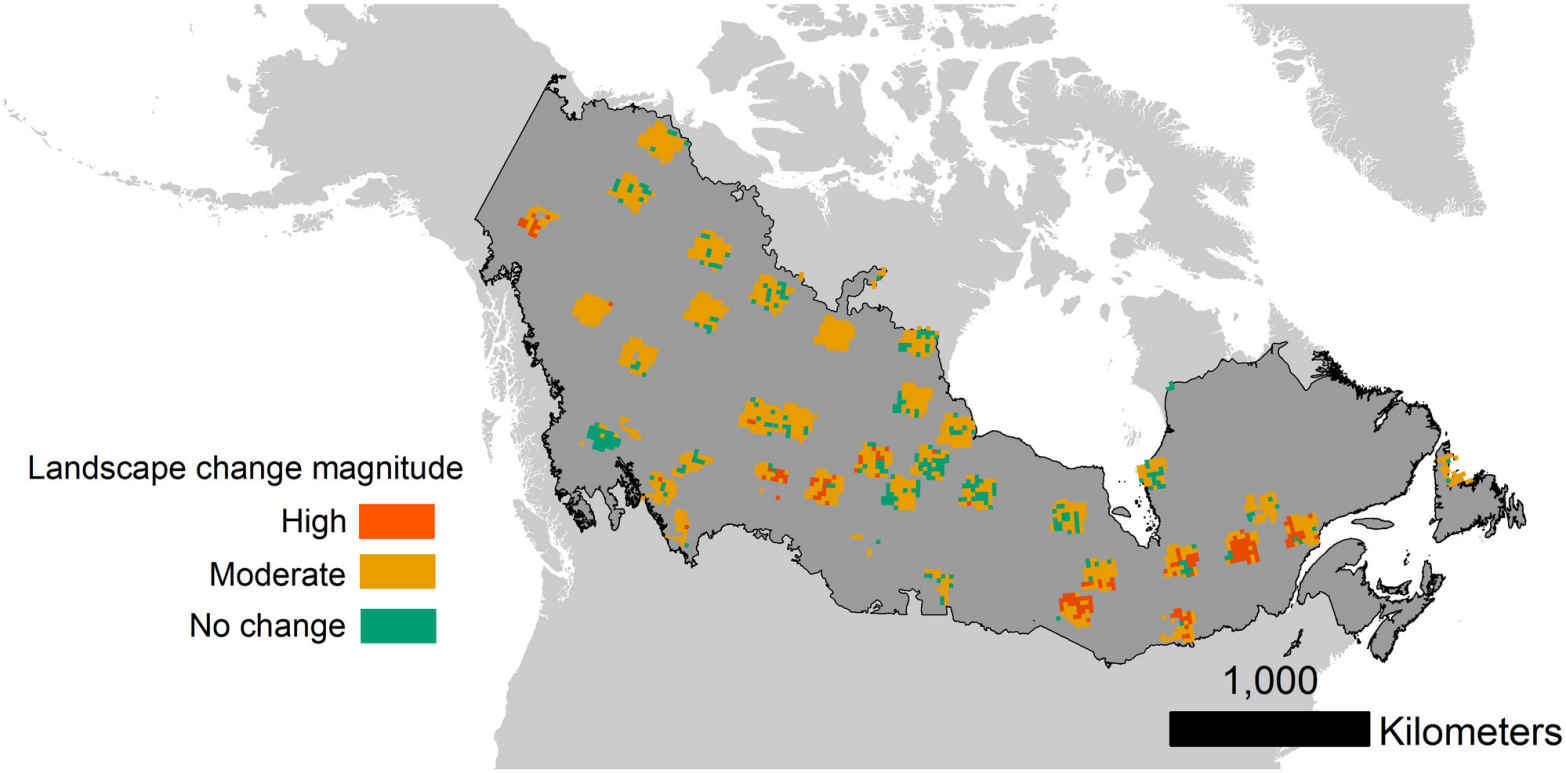
Locations of 50,000 ha landscapes classed by landscape magnitude change across the boreal forest zone of Canada.

**Fig 6:**
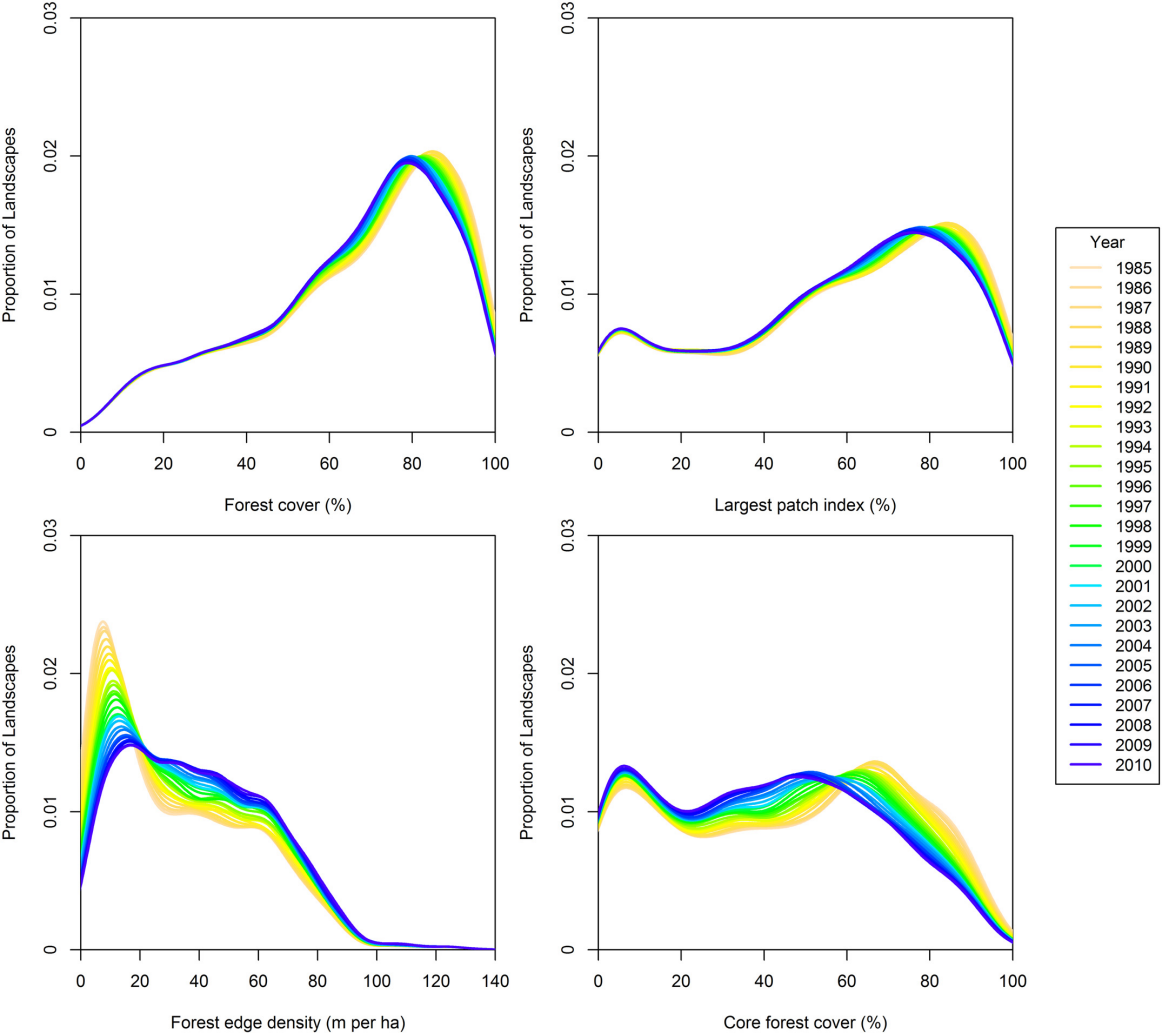
Local polynomial regression curves of landscape proportion by pattern index for the 50,000 ha landscape extent from 1985 to 2010. For all curves, *n* = 1,662 landscapes.

**Fig 7:**
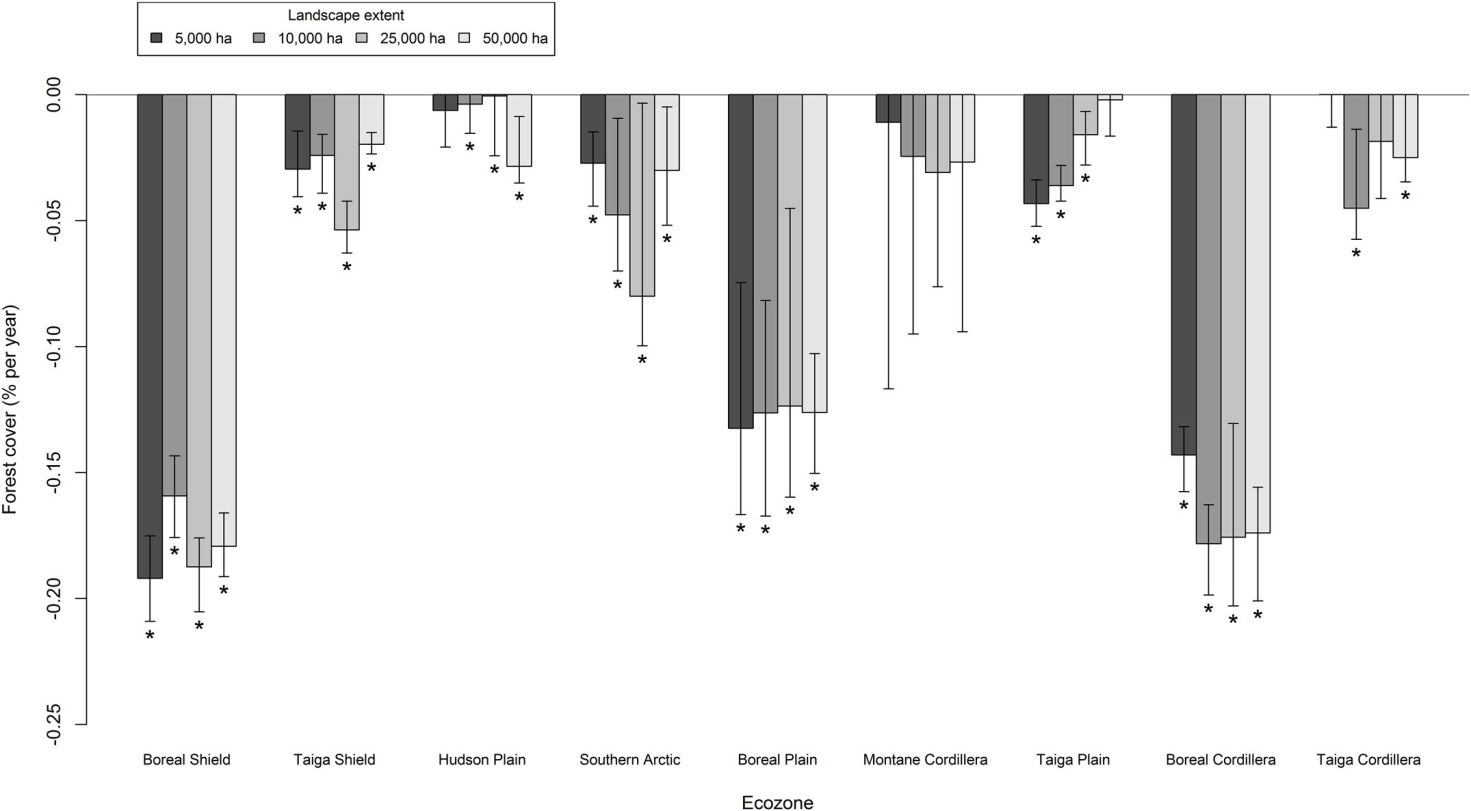
Median Theil-Sen slope of forest cover from 1985 to 2010 for each ecozone and landscape extent. Error bars indicate the upper and lower 95% confidence bounds. Asterisks (*) indicate significant (p < 0.05) monotonic trends from a Mann-Kendall test.

The majority of 50,000 ha landscapes had significant forest cover change. Approximately 74% (1,232) of 50,000 ha landscapes were characterized by moderate magnitude change and 8% (134) were characterized by high magnitude change. The classes of landscape pattern change were mapped across the boreal forest of Canada in Fig 5. The mode of forest cover for the 50,000 ha extent shifted over the observation period from approximately 85% in 1985 to 79% in 2010 (Fig 6). The median value of forest cover ranged from approximately 73% in 1985 to 70% in 2010 for the 50,000 ha extent and from 77% in 1985 to 74% in 2010 for the 5,000 ha extent. The Boreal Shield, Hudson Plain, Boreal Plain, Montane Cordillera, Taiga Plain, and Boreal Cordillera ecozones were the most forested with median forest cover greater than 50%. There was a significant negative trend over time in forest cover across most ecozones and extents (Fig 7). The most forested ecozones underwent the largest magnitude changes in forest cover in terms of the median Theil-Sen slope (Fig 7). The Montane Cordillera ecozone was the only ecozone that did not have a significant negative trend in forest cover and was also the most variable in terms of the error bars for the Theil-Sen slope estimate (Fig 7) despite being among the most forested ecozones. The Boreal Shield, Boreal Plain, and Boreal Cordillera ecozones had the greatest median forest cover change over the study period in excess of -0.1% · y^-1^ (Fig 7). Landscape extent had significant effects on observations of forest cover decline for the Boreal Shield, Taiga Shield, Taiga Plain, and Boreal Cordillera ecozones as determined from non-overlapping standard error bars for the change slopes (Fig 7). These results should be interpreted as differences in forest cover changes at specific scales. Forest cover declined at a significantly slower rate for 10,000 ha landscapes in the Boreal Shield and for 5,000 ha landscapes in the Boreal Cordillera as compared to other scales in those ecozones (Fig 7). In the Taiga Shield, forest cover declined at a significantly faster rate for 25,000 ha landscapes and for landscapes larger than 5,000 ha in the Taiga Plain ecozone (Fig 7). These results indicated scale-specific forest cover loss within some ecozones.

### Forest patch size

**Fig 8:**
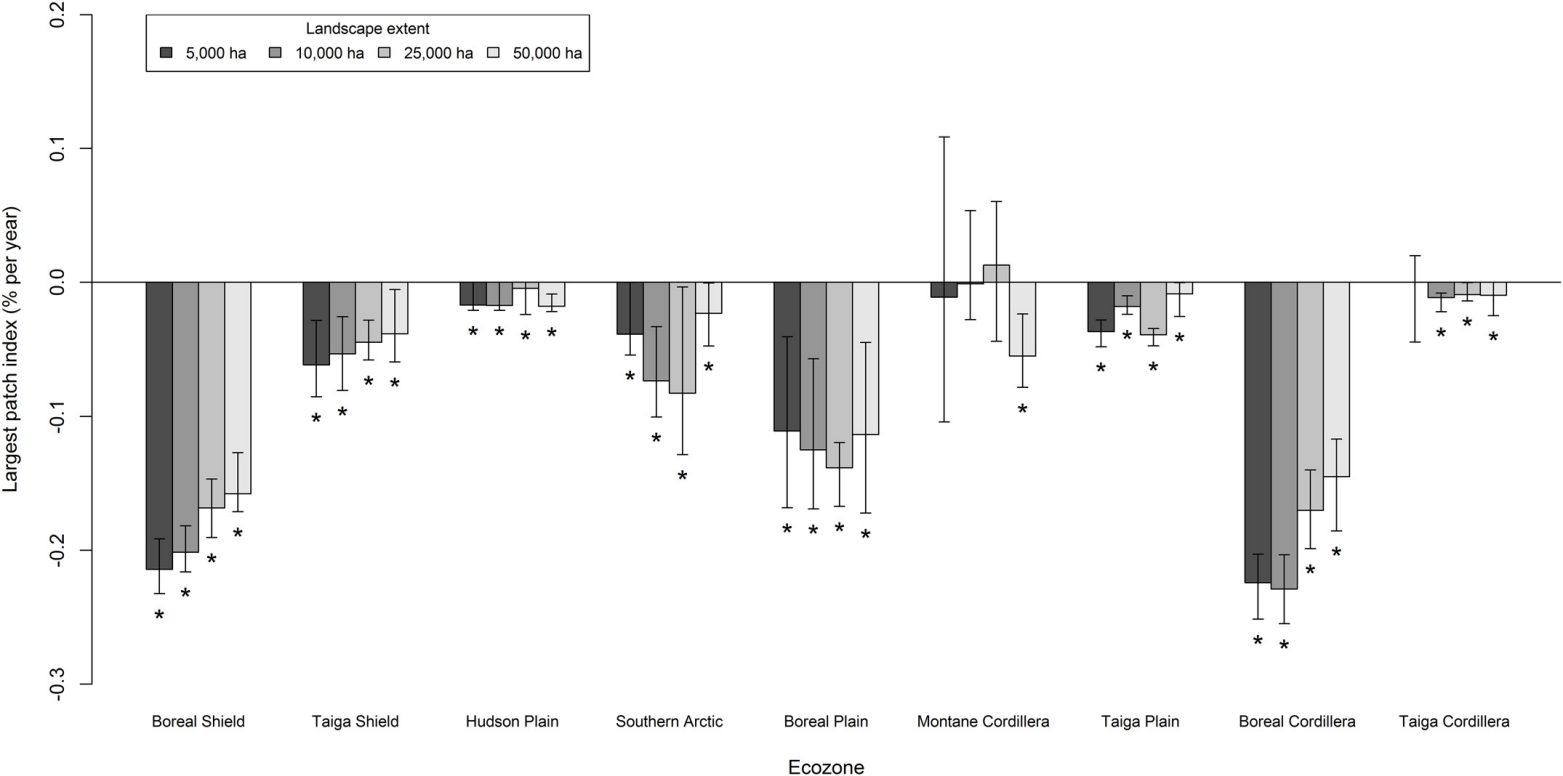
Median Theil-Sen slope of largest forest patch index from 1985 to 2010 for each ecozone and landscape extent. Error bars indicate the upper and lower 95% confidence bounds. Asterisks (*) indicate significant (p < 0.05) monotonic trends from a Mann-Kendall test.

As for forest cover, the largest forest patch index declined significantly over the study period for all landscape extents and ecozones (Fig 8). The greatest declines in the largest forest patch index were observed for the Boreal Shield, Boreal Plain, and Boreal Cordillera ecozones (Fig 8). Smaller landscape extents were associated with greater change rates for the Boreal Shield, Taiga Plain, and Boreal Cordillera ecozones (Fig 8). In 1985, the Boreal Plain ecozone had the greatest median value for the largest forest patch index (80%) and the Taiga Cordillera had the lowest median value (16%). The mode of largest forest patch index shifted over the study period from approximately 85% in 1985 to 76% in 2010 (Fig 6). The median value of largest forest patch index declined by approximately -0.09% · y^-1^ for all landscapes.

### Forest edge

**Fig 9:**
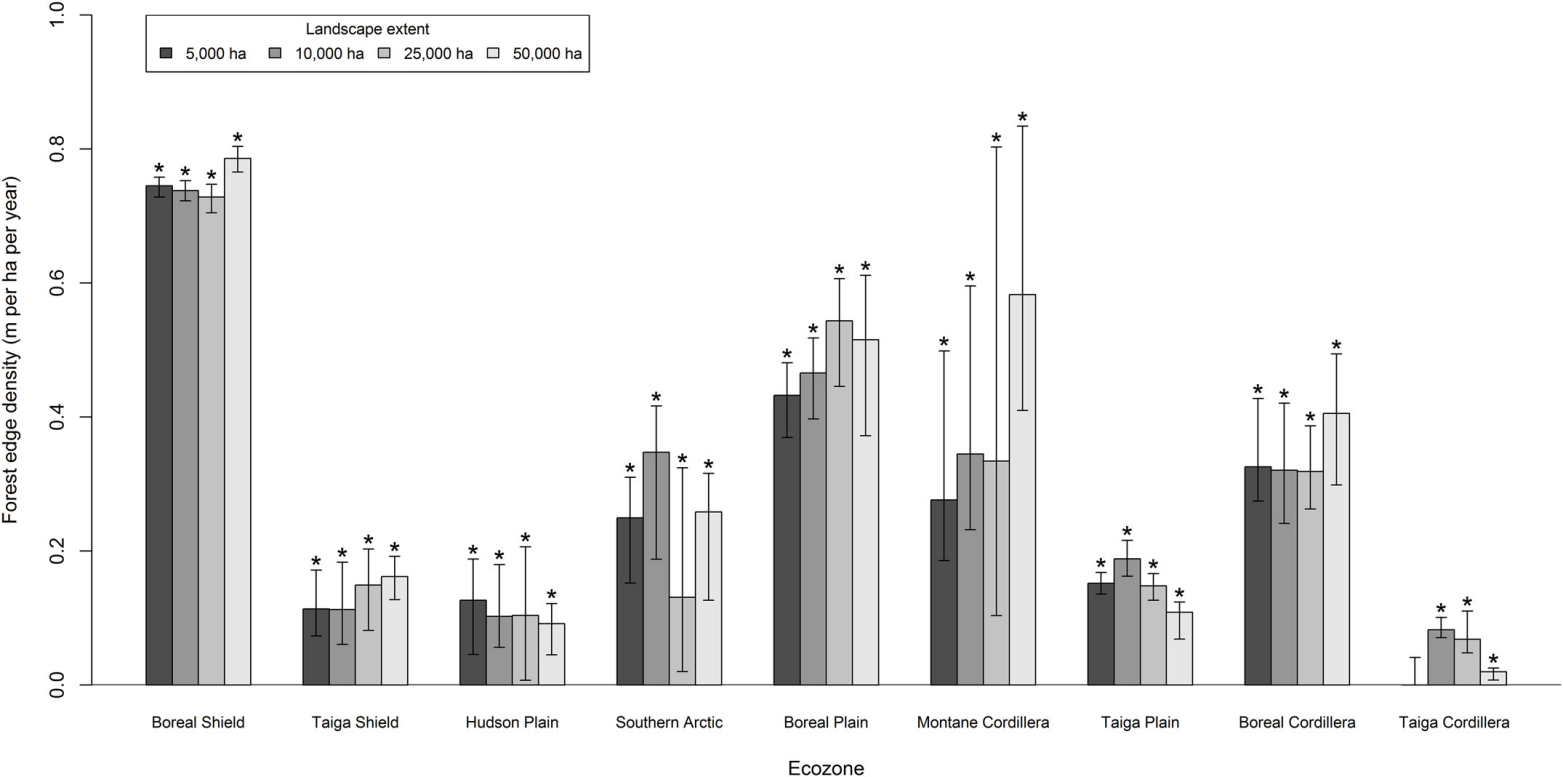
Median Theil-Sen slope of forest edge density from 1985 to 2010 for each ecozone and landscape extent. Error bars indicate the upper and lower 95% confidence bounds. Asterisks (*) indicate significant (p < 0.05) monotonic trends from a Mann-Kendall test.

Forest edge density increased for all landscape extents and ecozones over the study period (Fig 9). The median value of forest edge density increased at a rate of approximately 0.5 m · ha^-1^ · y^-1^ from 24 m · ha^-1^ to 38 m · ha^-1^ for all 50,000 ha landscapes. The most forested ecozones underwent the largest magnitude changes in forest edge density for 50,000 ha landscapes: 0.8 m · ha^-1^ · y^-1^ in the Boreal Shield; 0.5 m · ha^-1^ · y^-1^ in the Boreal Plain; and 0.4 m · ha^-1^ · y^-1^ in the Boreal Cordillera (Fig 9). Landscape extent significantly affected the Boreal Shield and Taiga Plain ecozones (Fig 9). The number of landscapes with low forest edge density (*i*.*e*., < 25 m · ha^-1^) declined while landscapes with intermediate levels of edge density increased over the study period for the 50,000 ha landscapes (Fig 6).

### Core forest cover

Core forest cover declined significantly for all landscape extents and ecozones. This trend was associated with declining forest cover in general and with the declining largest forest patch index. The Boreal Shield, Boreal Plain, and Boreal Cordillera ecozones had the largest magnitude changes in core forest cover per year (not shown). Generally, landscape extent had little effect on the observed change rates of core forest cover within ecozones. The number of 50,000 ha landscapes with high core forest cover (*i*.*e*., > 70%) declined over the study period by 41% from 413 in 1985 to 243 in 2010 (Fig 6). Within the ecozones, core forest cover of the 50,000 ha landscapes declined significantly at a rate of approximately -0.6% · y^-1^ in the Boreal Shield, -0.5% · y^-1^ in the Boreal Plain, -0.1% · y^-1^ in the Montane Cordillera, -0.1% · y^-1^ in the Taiga Plain, and -0.2% · y^-1^ in the Boreal Cordillera. The rate of core forest decline in the Boreal Shield (-0.6% · y^-1^) was significantly greater than all other ecozones except the Boreal Plain as determined from non-overlapping standard error bars for the change slopes. Core forest cover was bi-modal for the 50,000 ha extent in 1985 with the modes occurring at approximately 6% and 67% cover (Fig 6).

### Static boreal forest landscapes

**Fig 10:**
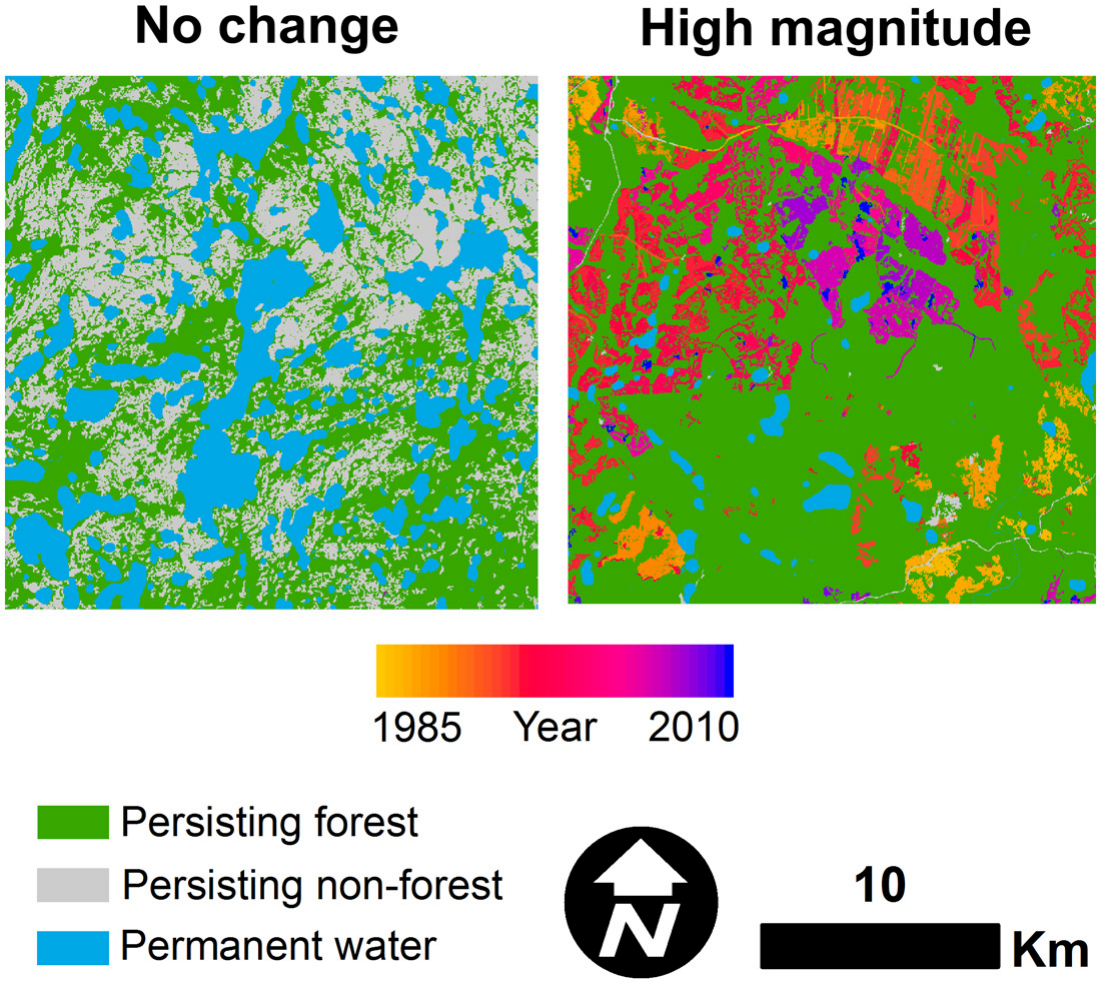
Examples of high magnitude change pattern for a 50,000 ha landscape in central Ontario (right) and a 50,000 ha no change landscape in Northwest Territories (left).

Approximately 18% of all 50,000 ha landscapes did not change as indicated by non-significant forest cover change slopes (*e*.*g*., less than 0.01% · y^-1^). These landscapes represented the control group for the pattern analysis and were distributed as follows among the ecozones (percentage expressed out of total landscapes sampled in that zone): 100 (18%) in the Boreal Shield; 54 (20%) in the Taiga Shield; 33 (16%) in the Boreal Plain; 30 (41%) in the Montane Cordillera; 56 (43%) in the Hudson Plain; 29 (14%) in the Taiga Plain; 8 (15%) in the Southern Artic; and 1 (33%) in the Taiga Cordillera. On average, these relatively stable landscapes were approximately 67% forested ($\tilde{X}$ = 71%) in 1985 with 12% of the landscape comprised of permanent water ($\tilde{X}$ = 8%). The median value of the largest patch index in the no change landscapes declined at a rate of -0.06% · y^-1^ from 65% to 63%. Forest edge density increased by approximately 0.4 m · ha^-1^ · y^-1^ from 27 m · ha^-1^ to 37 m · ha^-1^. Core forest cover declined at a rate of approximately -0.2% · y^-1^ from 47% to 41%. A comparison between examples of high magnitude forest pattern change and no change is shown in Fig 10.

### High magnitude changes in boreal forest landscapes

In total, 166 (10%) of the 50,000 ha landscapes were identified as showing high magnitude change, which were those ranked above the 90^th^ percentile in terms of forest cover change slope. High magnitude landscapes accounted for 126 (21%) of the landscapes in the Boreal Shield, 27 (12%) of landscapes in the Boreal Plain, and 12 (16%) of landscapes in the Boreal Cordillera. The median value of largest forest patch index declined by approximately -0.4% · y^-1^ from 83% to 73% for high magnitude change landscapes, which was four times greater than all landscapes. Also, the median value of forest edge density increased by approximately 1.4 m · ha^-1^ · y^-1^ from 10 m · ha^-1^ to 45 m · ha^-1^ for high magnitude change landscapes and core forest cover declined by approximately -1.1% · y^-1^ from 69% to 42%.

## Discussion

### Trends in historical boreal forest spatial patterns

The results presented here represent an initial assessment of landscape patterns of boreal forests across a sample of the available satellite archive. Generally, landscapes were characterized by moderate declines in forest cover and this was associated with declining largest patch size and core forest cover, and increasing forest edge density. Forest cover declined by approximately -0.06% · y^-1^, on average. The net decline in forest cover was a result of faster disturbance processes relative to forest recovery processes in the areas sampled. This net decline in forest cover over the 26-year period could be an early warning indicator of impending changes to landscape structure in the Canadian boreal forest [63]. Further research is needed to examine the possibility of critical transitions of landscape structure in the boreal system.

Due to the lag effect associated with observing vegetation recovery, recovery rates may be underestimated relative to disturbance rates over the same observation period [64]. This study showed that net forest cover declines resulted in increased edge density and loss of core forest cover. Provided sufficient time, many disturbed forests may be expected to fully recover, but this is not necessarily evident from our 26-year time series and binary forest/non-forest mapping approach. More recent disturbances in our time series have not had sufficient time to recover although those pixels are most likely in some phase of recovery that probably includes some degree of tree cover. Thus, our approach is limited by the time series of Landsat imagery and the pattern metrics should be interpreted as a snapshot in time that is expected to change in trajectory and magnitude as the time series is updated.

The general change trends noted here are consistent with what is known about resource development in boreal forests of Canada. For example, many of the largest declines in forest cover were observed for the Boreal Shield, Boreal Plain, and Boreal Cordillera ecozones. These are regions that are known to be impacted by extensive forest management and energy development [20,22,65]. The forest patterns of the eastern boreal landscapes of central Ontario and southern Quebec can be attributed to progressive dispersed clear cutting of the forest between 1985 and 2010 [66]. Similarly, the forest patterns of the western boreal landscapes of Alberta, Saskatchewan, and Manitoba can be attributed to road construction, forest harvesting, and energy development [67]. The Boreal Shield ecozone had the fewest landscapes that did not change over the study period relative to the total number of landscapes sampled in that zone (17%). By contrast, 76% of all high magnitude change landscapes occurred in the Boreal Shield, which represented 21% of all landscapes sampled in that zone. The significance of this finding should be underscored in terms of the relative importance of this ecozone which accounts for approximately one-third of all wood volume in Canada [68,69]. Although we examined different landscape extents, our results also agree with the findings of Wulder and colleagues [6] in terms of how the pattern indices vary by ecozone. For example, we found that forest cover generally increased as landscape extent decreased in all ecozones except the Taiga Cordillera ecozone [6]. The least forested ecozones (Taiga Cordillera, Taiga Plain, and Taiga Shield) had the lowest levels of forest edge density relative to the other ecozones *circa* 2000 [6].

Landscapes that did not change were associated with minimal disturbance, were approximately two-thirds forested, and were characterized by less than 40 m · ha^-1^ forest edge density on average. This level of forest edge density displayed characteristics of a threshold in landscape structure, above which core forest cover declined and largest forest patch index decreased (Fig 2d and 2e). Core forest cover and the largest forest patch comprised approximately 47% and 65%, respectively, of landscape area at the median in no change landscapes in 1985 and the annual change of forest cover was relatively small (less than 0.01% · y^-1^ on average). These results indicate that nearly one-fifth (18%) of all 50,000 ha landscapes are relatively intact, yet still undergo small changes in forest cover, probably due to localized disturbance. Moreover, some of the spatial patterns observed in no change landscapes could be attributed to lakes or other non-forested features of the landscape (Fig 10).

### Management implications

The pattern database results provide important context for forest management agencies committed to implementing ecosystem-based management (EBM) strategies. The specific trends noted over the last three decades demonstrate a moderate rate of forest pattern change in boreal forests. Although many jurisdictions across Canada have adopted EBM harvesting guidelines [70,71,72,73], the 26-year observation period was insufficient to fairly evaluate the implementation of recent EBM strategies, which continues to be in a transition period. The trajectories of the pattern metrics reflect a combination of factors including both “business-as-usual” forest harvesting and fire. Pattern trends in any given landscape may provide useful information to local expert forest managers since many of the landscapes in our sample intersect lands tenured for forest production as well as some of the most intensively managed forests. To some degree, the static boreal forest landscapes in our sample provide a range-of-variability of landscape patterns in the absence of recent disturbance. In addition to the 90^th^ percentile that we used to identify rapidly changing landscapes, pattern trends in the database may be stratified by any degree of disturbance, which enhances the flexibility of the database to be used to aid forest management planning.

### Limitations and considerations for future research

The methodology that we employed provided the capacity to observe the impacts of standing-replacing disturbance on boreal forest spatial patterns. The results demonstrate that the indices were robust at observing changes to boreal forest spatial patterns at a range of spatial scales. We focused primarily on forest patch area and edge indices due to their intuitive interpretation and relevance to boreal forest habitats. All of the indices that we used were invariant to landscape extent (*i*.*e*., scale) and were therefore well-suited to a cross-scale analysis of spatial patterns.

The conservative method we used to map forest cover changes likely resulted in underestimating the actual values of some of the pattern indices for a number of reasons. The VCT algorithm used to map the forest cover changes has been known to underestimate forest disturbance [37]. Reference data were insufficient to provide a systematic assessment of forest cover changes across the sampled portion of the boreal forest. However, we implemented a set of criteria for including forest cover changes in the annual maps such as persistence of change through multiple years, minimum mapping unit of approximately 1 ha, and large change magnitude. Thus, small, low severity, and ephemeral disturbances were not likely to be considered in the analysis, although we know that these changes also contribute to landscape change. As an example, our estimation of forest edge density was lower than what would be expected given the research of Wulder *et al*. [6]. These types of discrepancies are likely due to differences in data processing (*i*.*e*., VCT and BAP) as well as our objective of assessing large magnitude changes. Moreover, the pixel resolution and signal-to-noise ratio for the sensors onboard the Landsat satellites are not well-suited for detecting fine-scale disturbances. Therefore, our analysis is predominantly a stand-replacing representation of forest cover change of Canada’s boreal forests.

In the future, landscape pattern databases should be developed with higher thematic resolution and with other properties that permit cross-scale comparisons. In this study, the classes were simply *forest* and *non-forest* which were suitable for demonstrating the novel temporal property of the database while maintaining high class accuracy. Increasing the number of thematic land cover classes and, consequently, the number of potential combinations of land cover changes between classes increases the utility of such landscape pattern databases for applications in many more ecosystems, but also significantly increases computational complexity and introduces a greater propensity for map misclassification errors [43]. Additionally, further research is needed to develop database properties that enable cross-scale comparisons. Scaling has been identified as a key area for research in landscape ecology, specifically the selection of appropriate scale for a given investigation and understanding the relationship between pattern and scale [48]. Landscape pattern databases that have multi-scalar properties provide the means to understand the behavior of pattern indices at multiple scales and permit the selection of a scale that is appropriate for any given research objectives [74]. The development of future landscape pattern databases should maintain temporal and scaling properties while providing higher thematic resolutions for the broadest possible applications.

## Conclusions

The temporal information in the pattern database provided the opportunity to assess how landscape patterns of boreal forest cover changed with time. The method was useful for estimating changes to forested landscape structure over broad spatial and temporal scales. The nature of the pattern results is consistent with what is known about resource development in the Canadian boreal forest. Additionally, inherent variability in forest cover was observed for some landscapes in the absence of recent disturbance. The results suggest that the landscape pattern indicators were sufficiently sensitive to even small changes in forest cover. Databases like the one we employed provide range-of-variability measures that can aid future research in contextualizing the configuration and composition of boreal forest landscapes in space and time.

## Supporting Information

S1 Table Annualized pattern indices for 5,000 ha landscapes. (TAR)

S2 Table Annualized pattern indices for 10,000 ha landscapes. (TAR)

S3 Table Annualized pattern indices for 25,000 ha landscapes. (TAR)

S4 Table Annualized pattern indices for 50,000 ha landscapes. (TAR)
